# Evolving Masculinity Norms Shape Men’s Willingness to Intervene in Workplace Sexual Harassment

**DOI:** 10.1007/s11199-025-01635-w

**Published:** 2026-02-05

**Authors:** Giulia Valsecchi, Vincenzo Iacoviello, Juan Manuel Falomir-Pichastor

**Affiliations:** Faculty of Psychology and Educational Sciences, University of Geneva, FPSE, Uni Mail, Boulevard du Pont d’Arve 40, CH-1211 Geneva 4, Switzerland

**Keywords:** Bystander intervention, Workplace sexual harassment, Masculinity norms, Antifemininity, Men’s feminization, Inclusive masculinity, Sexual violence prevention

## Abstract

Recent shifts in masculinity norms have led many men to adopt roles traditionally associated with femininity. This research examined whether perceived changes in gender norms influence heterosexual men’s willingness to intervene in workplace sexual harassment. Across three experimental studies (*N* = 614) in the United Kingdom, United States, and Western Europe, masculinity norms were manipulated (perceived feminization vs. traditional masculinity) and antifemininity motivation was assessed. Participants then evaluated a workplace sexual harassment scenario and reported willingness to intervene. Results indicated that perceived feminization reduced willingness to intervene (Studies 1–3) and increased justification of harassment (Study 3) among men high in antifemininity motivation. These findings highlight the role of masculinity norms in shaping bystander intentions and suggest that shifts toward inclusive masculinity may have unintended consequences for prosocial responses to sexual harassment.

**Supplementary Information:**

The online version contains supplementary material available at https://doi.org/10.1007/s11199-025-01635-w.

Workplace sexual harassment—defined as unwelcome verbal, nonverbal, or physical sexual conduct—represents a widespread form of gender-based violence. It is particularly prevalent in male-dominated work environments, where such behavior is often normalized or overlooked (Handy, 2006; Ilies et al., 2003; Timmerman & Bajema, 1999; Willness et al., 2007). The consequences for victims are significant, including diminished job satisfaction, weakened organizational commitment, and adverse effects on both psychological and physical health (Willness et al., 2007). In response to the prevalence of workplace sexual harassment, research increasingly emphasizes the importance of preventive strategies, particularly bystander intervention (McDonald et al., 2016). Bystanders—individuals who witness or become aware of harassment—can either remain passive or actively support the victim (Banyard et al., 2005; Powell, 2011; Salin, 2011). The likelihood of intervention is shaped by both organizational culture (Jacobson & Eaton, 2018; Mainwaring et al., 2022) and individual factors, including age, previous experiences of victimization, and levels of self-efficacy (Brewster & Tucker, 2016; Mainwaring et al., 2022).

Among these individual-level determinants, gender emerges as a particularly influential factor. Research consistently shows that men are significantly less likely than women to intervene in incidents of workplace sexual harassment (Bennett et al., 2014; Burn, 2009; Franklin et al., 2020; Savage et al., 2017). This disparity is often attributed to gender role socialization and hegemonic masculinity norms, which idealize dominance, emotional restraint, and detachment (Casey & Ohler, 2012; Connell, 2005; Connell & Messerschmidt, 2005). As societal conceptions of gender continue to evolve (Thompson & Bennett, 2015), understanding how these shifts influence men's engagement as active bystanders is critical—not only for advancing intervention strategies but also for challenging entrenched norms that inhibit prosocial behavior in harassment contexts.

To explore this dynamic, the present research examined whether perceiving masculine norms as shifting toward more feminine traits—referred to here as the *perceived feminization of men’s norms*—affects heterosexual men’s willingness to intervene in workplace sexual harassment scenarios. Specifically, we investigated whether this effect is moderated by men’s motivation to uphold the antifemininity norm, a central tenet of traditional masculinity ideology.

## The Role of Traditional Masculinity in Bystander Intervention

Research consistently shows that men are less likely than women to engage in bystander intervention in incidents of sexual harassment and tend to hold less favorable attitudes toward such actions (Banyard & Moynihan, 2011; Burn, 2009). This gender gap is closely tied to traditional masculine norms—particularly the antifemininity norm—which discourages emotional expression, empathy, and behaviors perceived as “soft” or feminine, including prosocial action (Croft et al., 2020; Paterson & Mahalik, 2024). These norms not only diminish men’s perceived capacity and willingness to intervene (Connell & Messerschmidt, 2005) but also foster social pressures that discourage men from disrupting other men’s sexual pursuits or appearing emotionally invested (Carlson, 2008; Fabiano et al., 2003; Katz, 2006). While the protective impulse toward women—often framed as chivalry—has traditionally been viewed as a masculine trait, it is typically reserved for close personal relationships, such as family members. This selective expression reinforces the notion that intervention is not a general moral obligation, but rather a context-dependent duty (Good et al., 2018).

Recent findings further refine this understanding by showing that specific gender norms are responsible for the decreased likelihood of engaging in bystander behaviors (Baildon et al., 2025). Among men, stronger adherence to norms emphasizing dominance over women, emotional restraint, and heterosexual self-presentation predicts less bystander intervention, whereas risk-taking is associated with greater engagement in bystander intervention. For women, norms that restrict sexuality and prioritize romantic relationships tend to inhibit intervention, while valuing kindness and friendship enhances it. These patterns underscore antifemininity as a central barrier to prosocial action among men and highlight the importance of targeting specific normative beliefs rather than broad gender identities in intervention efforts.

Although traditional masculinity has long been viewed as an obstacle to bystander intervention, these findings call for a more nuanced understanding of its multiple dimensions. Specifically, they highlight the potential of alternative masculinities—those embracing emotional openness and relational responsibility—to promote accountability and encourage intervention in sexual harassment contexts (Stewart, 2014; Thompson & Bennett, 2015). The present research investigates how the perceived feminization of men’s norms influences men’s intentions to intervene as bystanders in hypothetical workplace sexual harassment scenarios.

## Perceived Feminization of Men’s Norms and its Impact on Men’s Bystander Intentions

Critical studies on men and masculinities engage with debates on the evolving nature of masculine roles and expressions (e.g., de Boise, 2022). Some scholars suggest that hybrid and more inclusive forms of masculinity are emerging, as men adopt behaviors traditionally associated with femininity (Christofidou, 2021; Wolfman et al., 2021). Research highlights men’s growing involvement in domestic responsibilities, childcare, and family life (Roberts, 2017; Warren, 2022), the adoption of gender-neutral parenting roles (Johansson & Andreasson, 2017), and greater emphasis on emotional expressiveness (Andreasson & Johansson, 2016; Eisen & Yamashita, 2019; Elliott, 2016; Wall et al., 2007).

Public opinion reflects this trend, with many men acknowledging that rigid, traditional gender stereotypes are restrictive and harmful (Flood, 2020). Supporting this view, a *GQ* magazine survey of 1,005 U.S. respondents found that 97% perceived changes in societal expectations of masculinity over the past decade, and nearly half of men (48%) reported feeling comfortable with these changes (GQ, 2019). We propose that such perceived feminization of men’s norms may influence men’s intentions to intervene as bystanders in workplace sexual harassment scenarios.

Drawing on social role theory (Diekman & Goodfriend, 2006; Eagly, 1987; see also gender schema theory; Bem, 1974) and precarious manhood theory (Vandello & Cohen, 2008; Vandello et al., 2013), we hypothesize that the perceived feminization of men’s norms can either increase (via conformity dynamics) or decrease (via resistance dynamics) men’s willingness to intervene. This dynamic may depend on men’s motivation to uphold the antifemininity norm. Below, we outline the conformity and resistance hypotheses and consider the potential moderating role of antifemininity motivation.

### Conformity Dynamics: How Perceived Feminization of Men’s Norms May Increase Men’s Intentions to Intervene

According to social role theory (Eagly & Wood, 2012), individuals associate traits with the social roles that different groups occupy. Traditionally, women have been associated with communal traits such as kindness and sensitivity, reflecting caregiving roles, whereas men have been linked to agentic traits like assertiveness and independence, reinforcing dominance in leadership and power-oriented domains (Bosak et al., 2018; Diekman & Eagly, 2000; Diekman & Goodfriend, 2006; Froehlich et al., 2020). When gender roles are perceived as shifting—for instance, when men increasingly take on caregiving responsibilities—stereotypical gender representations tend to diminish (Bosak et al., 2018).

Social role theory also helps explain why such shifts may encourage men to intervene in workplace sexual harassment scenarios. As men increasingly occupy roles associated with communal traits—such as emotional support, caregiving, and social responsibility—they may conform to emerging norms that explicitly promote empathy, accountability, and support for gender equality. These qualities, traditionally linked to femininity, are being redefined as integral to contemporary masculinity, contrasting with norms of emotional restraint, dominance, and antifemininity. Research shows that when men perceive gender roles as less rigid—such as when gender differences in agency are no longer salient—they adopt more egalitarian attitudes and behaviors (Kosakowska-Berezecka et al., 2016). Similarly, exposure to changing norms increases interest in counter-stereotypical roles and reduces adherence to hegemonic masculinity (Bosak et al., 2018; Cheng et al., 2020; Iacoviello et al., 2024).

In the context of workplace sexual harassment, bystander intervention may no longer be perceived as unmanly but instead as a socially endorsed behavior aligned with evolving conceptions of manhood. Thus, the *perceived feminization of men’s norms* may increase men’s intentions to intervene by aligning prosocial behavior with a broader, more inclusive understanding of masculinity.

### Resistance Dynamics: How Perceived Feminization of Men’s Norms May Decrease Men’s Intentions to Intervene

In contrast to conformity dynamics, precarious manhood theory (Vandello et al., 2008, 2013) suggests that the perceived feminization of men’s norms may elicit resistance among men. Precarious manhood theory suggests that manhood is a socially fragile status, one that requires constant validation and protection, making men particularly sensitive to perceived threats to their masculinity. When masculine boundaries become blurred—whether due to diminishing distinctions between male and female traits (Bosson & Michniewicz, 2013) or perceptions of men as increasingly feminized (Babl, 1979; Iacoviello et al., 2020)—men often exhibit defensive responses.

These defensive reactions are not merely symbolic; they manifest in behaviors that reinforce traditional masculine ideals, including physical aggression (Bosson et al., 2009), heightened drives for muscularity (Mills et al., 2023), and reduced willingness to confront sexual prejudice (Kroeper et al., 2014). Importantly, such responses are also linked to more favorable attitudes toward sexual violence (Vescio et al., 2023). Such responses are deeply rooted in traditional masculinity norms—particularly the antifemininity component—which emphasize dominance, emotional detachment, and sexual entitlement. By fostering these dispositions, these norms may diminish moral discomfort when witnessing harassment and, consequently, discourage intervention.

Thus, the perceived feminization of men’s norms— such as a growing emphasis on emotional expressiveness, vulnerability, and relational accountability—can be experienced by some men as a threat to their gender identity. Rather than embracing these evolving norms, they may react defensively, reaffirming traditional masculine ideals. Drawing on precarious manhood theory, we hypothesize that the perceived feminization of men’s norms may decrease men’s intentions to intervene as bystanders in workplace sexual harassment scenarios.

### The Moderating Role of Men’s Motivation to Uphold the Antifemininity Norm

Although social role theory and precarious manhood theory predict divergent responses to the perceived feminization of men’s norms, these perspectives may be reconciled by accounting for men’s attachment to traditional masculinity. Conformity to evolving norms is more likely among men who endorse less traditional masculinity (Valsecchi et al., 2023). Such men tend to reject stereotypically masculine traits (Babl, 1979) and report lower anxiety about being misclassified as gay (Borinca et al., 2021). In contrast, men more strongly committed to traditional masculinity often respond defensively—reinforcing masculine self-conceptions (Babl, 1979), expressing hostility toward feminist movements (Rivera-Rodriguez et al., 2022), derogating non-traditional men (Iacoviello et al., 2021), and showing heightened prejudice toward gay (Falomir-Pichastor et al., 2019; Valsecchi et al., 2022) and trans individuals (Burns et al., 2024).

In this study, we focus on endorsement of the antifemininity norm, a core dimension of traditional masculinity. This norm involves rejecting behaviors perceived as feminine—such as emotional vulnerability, empathy, and relational sensitivity—which are considered incompatible with dominant masculine ideals emphasizing toughness, control, and sexual assertiveness. Social pressures further discourage men from challenging other men’s sexual pursuits, reinforcing the notion that intervening in harassment contexts violates masculine expectations (Carlson, 2008; Fabiano et al., 2003; Katz, 2006).

Prosocial actions like bystander intervention often require emotional engagement and moral concern—traits traditionally associated with femininity. Prior research shows that adherence to antifemininity norms is linked to reduced empathy and greater tolerance for sexual violence (Leone & Parrott, 2019; Leone et al., 2016, 2017). Men who strongly endorse antifemininity are less likely to intervene, partly because such actions threaten their masculine identity and expose them to peer sanctions. Intervention aligns with communal values and may therefore be perceived as incompatible with the antifemininity mandate (Leone et al., 2016, 2017). Moreover, these men may interpret harassment as less serious or less deserving of intervention, consistent with norms that normalize male sexual entitlement (Leone & Parrott, 2019).

To test the causal role of antifemininity motivation, we assessed participants’ endorsement of the norm and experimentally manipulated their motivation to affirm it (e.g., Bosson et al., 2009; Wong & Wang, 2022). We propose that men’s motivation to uphold antifemininity—whether measured or induced—moderates’ responses to the perceived feminization of men’s norms. Specifically, men with a low motivation towards antifemininity should exhibit conformity effects, while those with a high motivation towards antifemininity are expected to respond defensively.

## Present Research

We conducted three experimental studies with heterosexual male participants to examine whether the perceived feminization of men’s norms influences their willingness to intervene as active bystanders in workplace sexual harassment scenarios. We also investigated whether this effect is moderated by motivation to uphold the antifemininity norm—conceptualized either as a dispositional trait (assessed in Studies 1 and 3) or as a contextually activated factor (manipulated in Study 2). The manipulation was based on prior evidence that lower perceived ingroup prototypicality heightens conformity to masculine norms, particularly antifemininity (Bosson et al., 2009).

Across all studies, participants were randomly assigned to one of two conditions: a *traditional masculinity* condition emphasizing continued endorsement of conventional masculine norms, or a *perceived feminization* condition highlighting shifts toward traditionally feminine roles and behaviors. Following the manipulation, participants read a hypothetical workplace sexual harassment scenario and reported their willingness to intervene.

We hypothesized that the perceived feminization of men’s norms would affect bystander intervention intentions depending on the antifemininity motivation. Specifically, participants with a low antifemininity motivation are expected to report greater willingness to intervene in the feminization condition than in the traditional masculinity condition (H1a: conformity dynamic). Conversely, participants with a high antifemininity motivation are expected to report lower willingness to intervene in the feminization condition than in the traditional masculinity condition (H1b: resistance dynamic).

All studies complied with APA ethical guidelines and were approved by the University Commission for Ethical Research at the University of Geneva. Participation was voluntary, and informed consent was obtained at the outset of each study. Due to the use of deception in the experimental manipulations, a second consent was obtained after full debriefing. This research was supported by the Swiss National Science Foundation (grant No. 00019_176080). 

## Study 1

In our initial study, we examined whether the perceived feminization of men’s norms influences men’s willingness to intervene in workplace sexual harassment scenarios, and whether this effect is moderated by antifemininity motivation. Participants’ motivation to uphold the antifemininity norm was assessed as an individual difference using self-report measures. They were then randomly assigned to either a *perceived feminization* or *traditional masculinity* condition, which manipulated perceptions of men’s gender norms. Following the manipulation, participants read a hypothetical workplace sexual harassment scenario and reported their willingness to intervene. We also included an exploratory measure of harassment perception to assess whether perceived feminization not only affects intervention intentions, but also the identification of harassment, as suggested by prior research (Cole et al., 2020; Rizzo et al., 2021).

## Method

### Participants and Design

Participants were self-identified heterosexual British men recruited via Prolific and compensated £1. The study employed a between-subjects design with two conditions: *perceived feminization of men’s norms* versus *traditional masculinity*. Participants were randomly assigned to one of the two conditions. Endorsement of the antifemininity norm (standardized) was included as a continuous moderator and assessed before the experimental manipulation.

To estimate sample size, we considered the design as a 2 × 2, following best practices for psychological research (Simmons et al., 2011, 2013), and aimed for at least 50 participants per cell. A total of 251 eligible participants completed the study. We excluded one for missing consent and six for reported inattention or dishonesty, resulting in a final sample of 244 participants (*M*_age_ = 37.84 years, *SD*_age_ = 12.64 years). A post-hoc sensitivity analysis using G*Power indicated that the study was adequately powered (power = .80, α = .05) to detect a small effect size (*f*^*2*^ = .02) in a multiple regression model with two main effects and one interaction. Most identified as White British (*n* = 217, 88.9%), with smaller proportions identifying as Asian British (*n* = 13, 5.3%), Black British (*n* = 7, 2.9%) and ‘Other’ (*n* = 7, 2.87%).

### Procedure and Materials

Participants were informed that the study aimed to gather opinions on gender-related issues. Demographic information was collected at the outset. All participants completed the survey in the same order: endorsement of the antifemininity norm, experimental manipulation, workplace sexual harassment scenario, and response measures. Unless otherwise specified, responses were recorded on 7-point Likert-type scales (1 = *Not at all / Strongly disagree*; 7 = *Absolutely / Strongly agree*). At the end of the study, participants were fully debriefed and asked to provide a second informed consent for data use due to the inclusion of deceptive elements.

#### Endorsement of the Antifemininity Norm

Antifemininity motivation was assessed using the Avoidance of Femininity (AF) subscale of the Male Role Norms Inventory–Short Form (MRNI-SF; Levant et al., 2013). The subscale includes three items: “Men should watch football games instead of soap operas,” "A man should prefer watching action movies to reading romantic novels," and "Boys should prefer to play with trucks rather than dolls.” Scores were averaged, with higher values indicating stronger endorsement of antifemininity (*M* = 2.87, *SD* = 1.53). Internal consistency was good (α = .86).

The MRNI-SF is a validated short form of the Male Role Norms Inventory–Revised (MRNI-R), capturing seven dimensions of traditional masculinity ideology: Avoidance of Femininity, Negativity toward Sexual Minorities, Self-Reliance through Mechanical Skills, Toughness, Dominance, Importance of Sex, and Restrictive Emotionality. The MRNI-SF was created by selecting the three highest-loading items from each MRNI-R subscale (Levant et al., 2010). Structural validity has been supported through factor analyses (Levant et al., 2013, 2016; McDermott et al., 2017), and convergent validity is evidenced by correlations with related measures such as the CMNI-46 (Parent & Moradi, 2009) and GRCS-SF (Wester et al., 2012). Although no direct equivalent exists for the AF subscale, exploratory analyses show significant associations with multiple CMNI-46 and GRCS-SF dimensions. Prior studies report internal consistency of α = .89 for the AF subscale (Levant et al., 2016).

#### Experimental Manipulation

Men’s gender norms were experimentally manipulated using a procedure adapted from Iacoviello et al. (2021). Participants read a bogus article summarizing findings from a purported longitudinal study conducted at a prestigious British university, which examined men’s personalities and behaviors from 1957 to 2017. In the perceived feminization condition, the article stated that “men tend to become more feminine over time and that the distinction between ‘being a man’ and ‘being a woman’ tends to disappear.” In the traditional masculinity condition, the article asserted that “men are just as masculine as ever and that the distinction between ‘being a man’ and ‘being a woman’ remains fundamental.” To strengthen the manipulation, participants were asked to provide a personal example illustrating the article’s claims.

#### Manipulation Check

Perceptions of men’s gender norms were measured using a three-item scale adapted from Iacoviello et al. (2021). Participants indicated their agreement with statements such as: “Men’s way of being has changed in recent years,” “Men are becoming more feminine over time,” and “Today, men are more feminine than ever.” Responses were averaged to form a composite score, with higher values indicating stronger perceptions of the feminization of masculine norms (*M* = 4.87, *SD* = 1.24). Internal consistency was acceptable (α = .79).

#### Sexual Harassment Scenario

Participants evaluated a hypothetical workplace sexual harassment scenario in which a male supervisor harassed a female subordinate. They were asked to imagine themselves as witnesses, positioned hierarchically below the perpetrator. This framing aligns with empirical evidence indicating that most workplace sexual harassment incidents involve perpetrators in positions of authority, highlighting the influence of power dynamics on bystander behavior (McDonald et al., 2016). All participants read the following scenario:

*Imagine that you are a physician on your rounds at the hospital. As you walk through the corridors, you overhear a conversation between your boss and a female colleague. You learn that your boss has invited her on a date, but she politely declines. He continues to insist, while she appears visibly uncomfortable. After she repeatedly rejects his invitation, he concludes the conversation by saying, ‘Don’t worry, it’s not like I’m going to fire you over this.’ With a forced smile, she eventually leaves the room.*

#### Perception of Sexual Harassment

To assess participants’ recognition of the scenario as sexual harassment, we used a single-item measure developed for this study: *“Do you consider this to be an instance of sexual harassment?”*, with higher values indicating stronger recognition of the incident as sexual harassment (*M* = 5.65, *SD* = 1.51).

#### Willingness to Intervene

Willingness to intervene was assessed with a 5-item scale developed for this study, reflecting direct, indirect, and passive intervention strategies consistent with prior research (Banyard et al., 2004). Items included: "I would contact human resources and report the incident," "I would talk to my boss about the incident," "I would go see my female colleague and ask if she wants to talk about the incident," "I would coordinate a workplace roundtable discussion addressing sexual harassment and sexism," and “I would refrain from taking any specific action and pretend I haven't witnessed anything" (reversed item)*.*

An exploratory factor analysis supported a one-factor solution for the scale. Full results, including parallel analysis and MAP test outcomes, are presented in Tables S1 to S3 in the online supplement. Reliability for scores on this scale was estimated at α = .74 in our sample. Items were averaged, with higher scores indicating greater willingness to intervene (*M* = 4.05, *SD* = 1.18). A one-sample *t*-test revealed that the mean score did not significantly differ from the scale midpoint (4), *t*(243) = 0.70, *p* = .509, *d* = 0.04, suggesting that, on average, participants reported moderate levels of willingness to intervene.

## Results

Descriptive statistics and bivariate correlations among the key study variables are reported in Table 1. To examine the effects of the experimental manipulation and individual differences, we conducted multiple regression analyses predicting both the manipulation check and the dependent variables. Predictor variables included men’s gender norm (−1 = traditional masculinity, + 1 = perceived feminization), standardized scores of antifemininity endorsement, and the interaction between these two variables.

**Table 1:** Descriptive Statistics and Intercorrelations Between Variables (Study 1)

Variable	*M*	*SD*	1	2	3
1. Endorsement of the antifemininity norm	2.87	1.53	–		
2. Perception of sexual harassment	5.65	1.51	-.20*	–	
3. Willingness to intervene	3.89	0.86	-.16*	.29*	–

*Note*. *p < .01

### Manipulation Check

The analysis revealed a significant main effect of the gender norm manipulation, *B* = 0.39, *t*(240) = 5.11, *p* < .001, 95% CI [0.24, 0.54], *η*^2^_p_ = .10. As expected, participants in the perceived feminization condition (*M* = 5.24, *SE* = 0.11) perceived men as more feminine than did those in the traditional masculinity condition (*M* = 4.47, *SE* = 0.11). No other effects were significant (*ps* > .407).

### Perception of Sexual Harassment

There was a significant main effect of antifemininity endorsement, *B* = −0.30, *t*(240) = −3.13, *p* = .002, 95% CI [−0.49, −0.11], *η*^2^_p_ = .04. Stronger endorsement of the antifemininity norm was associated with lower recognition of the scenario as sexual harassment. No other effects were significant (*ps* > .690).

### Willingness to Intervene

**Fig. 1:**
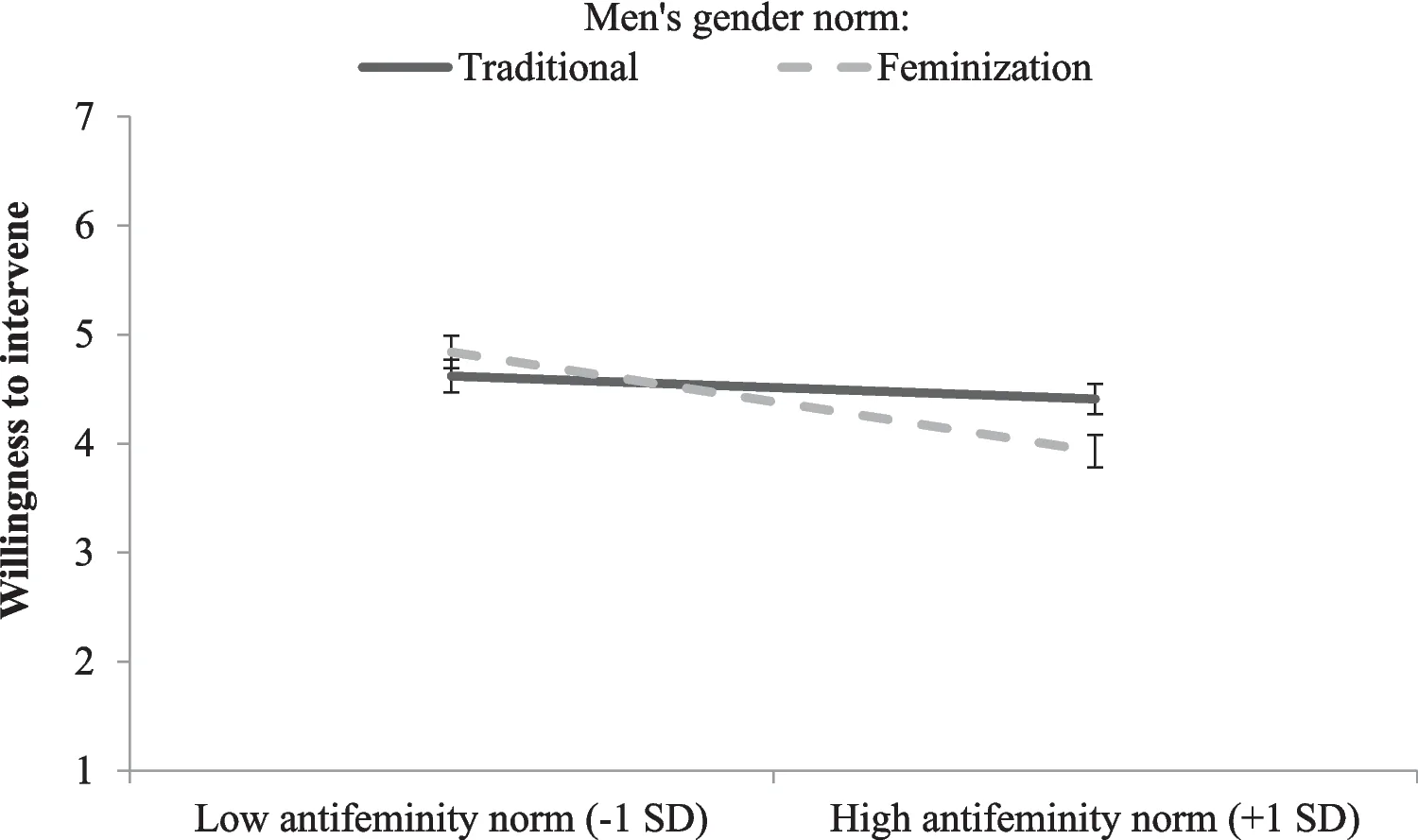
Willingness to intervene as a function of men’s gender norms and endorsement of the antifemininity norm (Study 1). Error bars are standard errors

Endorsement of the antifemininity norm significantly predicted willingness to intervene, *B* = −0.28, *t*(240) = −3.80, *p* < .001, 95% CI [−0.43, −0.14], *η*^2^_p_ = .06. Participants who more strongly endorsed this nom were less willing to intervene. The main effect of the manipulation was not significant, *B* = −0.06, *t*(240) = −0.84, *p* = .400, 95% CI [−0.21, 0.08], *η*^2^_p_ < .01, indicating that the manipulation alone did not influence participants’ intentions to intervene. However, the interaction between men’s gender norms and antifemininity was significant, *B* = −0.18, *t*(240) = −2.37, *p* = .019, 95% CI [−0.32, −0.03], *η*^2^_p_ = .02 (see Fig. 1). Simple slope analyses (Preacher et al., 2006) indicated that, consistent with the resistance hypothesis (H1b), participants high in antifemininity reported lower willingness to intervene in the feminization condition (*M* = 3.93, *SE* = 0.15) than in the traditional condition (*M* = 4.41, *SE* = 0.14), *B* = −0.24, *t*(240) = −2.28, *p* = .024, 95% CI [−0.44, −0.03], *η*^2^_p_ = .02. Thus, perceived feminization reduced bystander intention among individuals strongly endorsing antifemininity. Conversely, the conformity hypothesis (H1a) was not supported: among participants low in antifemininity, willingness to intervene did not differ between conditions, *B* = 0.11, *t*(240) = 1.09, *p* = .277, 95% CI [−0.09, 0.32], *η*^2^ₚ = .01.

## Discussion

The results showed that men's willingness to intervene as active bystanders in workplace sexual harassment incidents is shaped by their perceptions of contemporary masculine norms. Specifically, the findings supported the resistance hypothesis (H1b): Among men with strong antifemininity beliefs, exposure to information suggesting that men are becoming more feminine decreased their willingness to intervene. This suggests that perceived shifts away from traditional masculinity may provoke defensive reactions among those invested in maintaining rigid gender boundaries, thereby diminishing their likelihood of engaging in prosocial bystander behavior. In contrast, the conformity hypothesis (H1a) was not supported.

Beyond intervention intention, the study also revealed that antifemininity endorsement negatively influenced recognition of sexual harassment. That is, men higher in antifemininity were less likely to perceive the scenario as harassment. This extends prior research (Carlson, 2008; Leone & Parrott, 2019; Leone et al., 2016, 2017) by showing that antifemininity not only suppresses intervention intentions but also shapes cognitive appraisals of what constitutes sexual harassment. Importantly, while antifemininity endorsement predicted recognition, the gender norm manipulation itself did not, suggesting that individual differences may play a stronger role than contextual framing in shaping these judgments.

While Study 1 conceptualized the motivation to uphold antifemininity as a dispositional difference, prior research (Wong & Wang, 2022) suggests that this motivation can also be contextually activated. Accordingly, Study 2 employed an experimental procedure to examine whether situational cues could elicit patterns of resistance or conformity in response to perceived shifts in gender norms.

## Study 2

Study 2 was designed to test our hypotheses by experimentally activating men’s motivation to uphold the antifemininity norm, rather than conceptualizing it as a dispositional difference. Drawing on research showing that threats to ingroup prototypicality can heighten conformity to traditional gender norms (Bosson et al., 2009; Vandello et al., 2008), we used a false feedback paradigm to manipulate this motivation. Following established procedures (e.g., Glick et al., 2007; Maass et al., 2003), male participants first completed a personality test and then received fictitious feedback indicating either a high level of masculinity (prototypicality affirmation condition) or a high level of femininity (prototypicality threat condition). This manipulation was designed to either affirm or challenge participants perceived alignment with men’s traditional gender norms, thereby increasing or decreasing their motivation to distance themselves from femininity. As in Study 1, participants were exposed to the gender norm manipulation (perceived feminization vs. traditional masculinity) before evaluating the workplace sexual harassment scenario. We assessed two primary outcomes: participants’ recognition of the scenario as sexual harassment and their willingness to intervene as active bystanders.

## Method

### Participants and Design

Self-identified heterosexual men residing in the United States were recruited via MTurk and compensated $0.90 for their participation. Participants were randomly assigned to one of four experimental conditions in a 2 × 2 between-subjects design, crossing the gender norm manipulation (perceived feminization vs. traditional masculinity) with the gender prototypicality feedback (threat vs. affirmation). The study materials were completed in a fixed order, with demographic information collected at the end. Participants were then fully debriefed and thanked for their participation. 

Following recommendations for detecting interaction effects (e.g., Simmons et al., 2011), we aimed to recruit a minimum of 50 participants per condition, resulting in a target sample size of 200. A total of 218 eligible participants completed the study. One was excluded for not providing consent, yielding a final sample of 217 men (*M* = 39.53 years, *SD* = 11.78). A post hoc sensitivity analysis conducted using G*Power indicated that *N *the study was adequately powered (power = 80%, α = .05) to detect a small effect size (*f*^*2*^ = 0.03) in a multiple regression model with two main effects and one interaction. The majority of participants identified as White American (*n* = 163, 75.1%), followed by Latino (*n* = 19, 8.8%), Asian American (*n* = 17, 7.8%), African American (*n* = 14, 6.5%), Native American (*n* = 1, 0.5%), and “Other” (*n* = 3, 1.4%).

### Procedure and Materials

Except for the gender prototypicality manipulation, the procedure replicated that of Study 1, and all measures remained unchanged. Participants first read an excerpted article designed to manipulate perceptions of men’s gender norm. They then completed a personality test and received false feedback indicating either a high score on masculinity (prototypicality affirmation) or femininity (prototypicality threat).

Following the manipulations, participants completed a series of measures in a fixed order. These included felt threat, serving as a manipulation check for gender prototypicality (*M* = 3.45, *SD* = 1.70, α = .94); perception of men’s gender norms, serving as a manipulation check for the gender norm manipulation (*M* = 4.43, *SD* = 1.77, α = .94); perception of sexual harassment (*M* = 5.52, *SD* = 1.63); and willingness to intervene as active bystanders (*M* = 4.09, *SD* = 1.48, α = .82).

Participants’ willingness to intervene did not significantly differ from the scale midpoint (4), *t*(216) = 0.94, *p* = .345, *d* = 0.06. An exploratory factor analysis supported a one-factor solution for the scale. Full results, including parallel analysis and MAP test outcomes, are presented in Tables S4 to S6 in the online supplement.

#### Gender Prototypicality Manipulation

We implemented a gender prototypicality manipulation based on procedures from Maass et al., (2003; Study 2) and Glick et al. (2007). Participants were asked to evaluate how well 10 stereotypically masculine traits (e.g., independent, dominant) and 10 stereotypically feminine traits (e.g., nurturing, likes children), drawn from Bem’s (1974) Sex Role Inventory, described themselves. Participants were then randomly assigned to one of two experimental conditions. In the affirmation condition, they were told their personality was predominantly masculine. In the threat condition, they were told it was predominantly feminine. To enhance credibility, participants were informed that the test incorporated reaction time measurements to provide a more accurate evaluation of their "true personality."

#### Perceived Threat

To verify the effectiveness of the gender prototypicality manipulation, we assessed participants perceived threat following the feedback. Emotional responses to the feedback were measured using a six-item scale adapted from Schmitt & Branscombe (2001). Sample items included “I am disappointed about my score” and “My score put me in a good mood” (reverse-coded). Responses were averaged to create a composite threat index, with higher scores reflecting greater perceived threat (*M* = 3.45, *SD* = 1.70). The original scale demonstrated acceptable internal consistency (α = .82), and reliability in our sample was very good (α = .94).

## Results

Descriptive statistics and bivariate correlations among the key study variables are reported in Table 2. To examine the effects of the experimental manipulations, we conducted multiple regression analyses predicting both the manipulation checks and the dependent variables. Predictors included men’s gender norm (−1 = traditional masculinity, + 1 = perceived feminization), gender prototypicality feedback (−1 = affirmation, + 1 = threat), and their interaction.

**Table 2:** Descriptive Statistics and Intercorrelations Between Variables (Study 2)

Variable	*M*	*SD*	1	2	3
1. Felt threat	3.45	1.70	–		
2. Perception of sexual harassment	5.52	1.63	-.14*	–	
3. Willingness to intervene	3.76	1.01	-.11	.37**	–

*Note*. *p < .05, **p < .01

### Manipulation Checks

Manipulation checks confirmed the effectiveness of both manipulations. For the gender norm manipulation, participants in the feminization condition (*M* = 5.13, *SE* = 0.16) were significantly more likely to perceive men as becoming more feminine than those in the traditional masculinity condition (*M* = 3.72, *SE* = 0.16), *B* = 0.71, *t*(213) = 6.40, *p* < .001, 95% CI [0.49, 0.92], *η*^2^ₚ = .16. Similarly, the gender prototypicality manipulation yielded a significant main effect, *B* = 1.02, *t*(213) = 8.60, *p* < .001, 95% CI [0.79, 1.26], *η*^2^ₚ = .26, with participants in the threat condition (*M* = 4.09, *SE* = 0.17) reporting greater threat than those in the affirmation condition (*M* = 2.04, *SE* = 0.17). No other effects were significant (*ps* > .645).

### Perception of Sexual Harassment

The analysis revealed a significant main effect of gender prototypicality manipulation, *B* = −0.25, *t*(211) = −2.24, *p* = .026, 95% CI [−0.47, −0.03], *η*^2^_p_ = .02. Participants in the threat condition (*M* = 5.27, *SD* = 1.72) were less likely to recognize the scenario as sexual harassment than those in the affirmation condition (*M* = 5.77, *SD* = 1.15). No other effects were significant (*ps* > .561).

#### Willingness to Intervene

The linear regression analysis revealed no significant main effects on willingness to intervene (*ps* > .402). The interaction between men’s gender norm and gender prototypicality was not statistically significant, *B* = −0.17, *t*(213) = −1.70, *p* = .090, 95% CI [−0.37, 0.03], *η*^2^ₚ = .01. However, given that the interaction was close to the significance threshold, we decided to conduct simple slopes analyses for exploratory purposes. Results revealed that in the threat condition, participants exposed to the feminization condition (*M* = 3.72, *SE* = 0.20) were marginally less willing to intervene than those exposed to the traditional condition (*M* = 4.27, *SE* = 0.20), *B* = −0.27, *t*(213) = −1.94, *p* = .054, 95% CI [−0.55, 0.01], *η*^2^ₚ = .02. This difference did not reach conventional levels of statistical significance. In the affirmation condition, willingness to intervene did not differ between the feminization (*M* = 4.25, *SE* = 0.20) and traditional masculinity conditions (*M* = 4.12, *SE* = 0.20), *B* = 0.07, *t*(213) = 0.47, *p* = .636, 95% CI [−0.55, 0.01], *η*^2^ₚ < .01.

## Discussion

Although not statistically significant, the pattern of results in Study 2 was consistent with those observed in Study 1. The predicted interaction effect did not reach conventional significance levels. However, exploratory simple slope analyses suggested that participants in the gender prototypicality threat condition—reflecting stronger endorsement of traditional masculinity—may have been less willing to intervene when exposed to feminization; nevertheless, no clear support was observed for the resistance hypothesis (H1b).

Study 2 also indicated that while the manipulation of men’s gender norms did not significantly affect harassment recognition, the manipulation of gender prototypicality did. Consistent with Study 1, participants in the threat condition were less likely to recognize the scenario as sexual harassment than those in the affirmation condition, suggesting a potential link between antifemininity motivation and reduced recognition.

## Study 3

Study 3 was designed to further examine hypotheses H1a and H1b in light of the mixed findings from Studies 1 and 2. Study 1 revealed a significant interaction between men’s gender norm manipulation and antifemininity motivation on willingness to intervene. Although Study 2 did not replicate this interaction, the pattern of results was similar to that observed in Study 1. Additionally, both studies indicated that the manipulation of men’s gender norms did not significantly influence participants’ recognition of sexual harassment.

Study 3 therefore aimed to examine whether shifting masculine norms influence the legitimation of sexual harassment. Importantly, individuals may recognize an event as sexual harassment yet still attempt to legitimize it. To address this, we introduced a measure of harassment legitimation, conceptually inspired by rape myth endorsement scales, which assesses agreement with false societal beliefs that justify or minimize male sexual aggression against women (Lonsway & Fitzgerald, 1995). Such myths—e.g., the belief that women “ask for rape” or that rape results from uncontrollable male desire—serve to shift blame from perpetrator to victim (Chapleau, 2010; Gray, 2006; Payne et al., 1999). Prior research has shown that men, especially those strongly endorsing traditional masculinity norms, are more likely than women to engage in victim-blaming (Angelone et al., 2018; Bell et al., 1994; Schneider et al., 2009).

We hypothesized that exposure to the feminization condition would increase harassment legitimation among men high in antifemininity motivation (resistance hypothesis, H2b). Although previous studies did not support the conformity hypothesis (H2a) among men low in antifemininity motivation, we retained this prediction for consistency: in this group, the feminization condition was expected to reduce legitimation of workplace sexual harassment compared to the traditional masculinity condition. To test these hypotheses, we replicated the methodology of Study 1, assessing antifemininity endorsement, manipulating men’s gender norms, and measuring both willingness to intervene and harassment legitimation. Minor refinements were made to the intervention measure.

## Method

### Participants and Design

This study was conducted as part of a student research internship. Participants were recruited from public spaces and via social media networks in Geneva and did not receive compensation. Only self-identified heterosexual men were eligible to participate.

Following the design of Study 1, Study 3 used a between-subjects design with two experimental conditions (men’s gender norm: perceived feminization vs. traditional masculinity). Endorsement of the antifemininity norm (standardized scores) was included as a continuous moderator. Participants were randomly assigned to one of the two experimental conditions.

To ensure adequate statistical power, we followed recommendations to recruit at least 50 participants per condition as in previous studies. Due to time constraints associated with the internship, data collection was limited to three weeks. A total of 162 eligible heterosexual men were recruited. After excluding five participants who withheld consent and four who reported inattentiveness or dishonesty, the final sample consisted of 153 men, with a mean age of 26.62 years (*SD* = 10.19). A post-hoc sensitivity analysis conducted using G*Power (multiple linear regression model, power = .80, α = .05) indicated that the study was adequately powered to detect a small effect size (*f*^2^ = 0.04). Of the 153 participants, 73 (49.02%) reported Swiss nationality, whereas 78 (50.98%) reported another nationality.

### Procedure and Materials

The procedure closely mirrored that of Study 1. Participants first completed the same measure of antifemininity endorsement (*M* = 2.36, *SD* = 1.41, α = .82). However, unlike Study 1, the original measure of sexual harassment perception was substituted with a scale assessing harassment legitimation.

#### Willingness to Intervene

The original passive bystander item (“I would do nothing in particular and just pretend I have seen nothing”) was ambiguous regarding the witness’s intentions. To address this, two distinct items were introduced: one representing benevolent inaction (“I would do nothing in particular, but remain vigilant of the situation, ensuring I am present if the situation recurs”) and one representing hostile inaction (“I would do nothing in particular, but feel sympathy for the perpetrator, since nowadays all women say no”). The former was retained within the willingness-to-intervene scale, whereas the latter was included in the harassment legitimation measure. Additionally, the item “I would talk to my boss about the incident” was reworded for clarity as “I would talk to my boss about the incident and firmly remind him of the meaning of consent.” The remaining three original items were unchanged.

In the current sample, internal consistency for the full scale was initially low (α = .41). Removing the passive benevolent inaction item—“I would do nothing in particular, but remain vigilant of the situation, ensuring I am present …”—improved reliability to α = .67, suggesting that its passive framing may have reduced coherence with the other items. The final scale included four items, with higher scores indicating greater willingness to intervene (*M* = 4.49, *SD* = 1.20). Participants willingness to intervene was significantly higher than the scale midpoint (4), *t*(161) = 5.03, *p* < .001, *d* = 0.41, indicating relatively high levels of willingness to intervene. An exploratory factor analysis supported a one-factor solution for the scale. Full results, including parallel analysis and MAP test outcomes, are presented in Tables S7–S9 in the online supplement.

#### Legitimation of Sexual Harassment

To assess legitimation of harassment, participants rated agreement with two statements: "I would do nothing in particular, but feel sympathy for the perpetrator, since nowadays all women say 'no'" and "I would go see the female colleague to tell her that today, a man who flirts with a woman is immediately perceived as a harasser." The two items were significantly correlated, *r*(153) = .31, *p* < .001. Responses were averaged, with higher values indicating greater legitimation of sexual harassment (*M* = 1.79, *SD* = 0.99).

## Results

Descriptive statistics and bivariate correlations for key study variables are presented in Table 3. As in Study 1, we conducted multiple regression analyses predicting both the manipulation check and the dependent variables. Predictor variables included men’s gender norm (−1 = traditional masculinity, + 1 = perceived feminization), standardized scores of antifemininity endorsement, and the interaction between these two variables.

**Table 3:** Descriptive Statistics and Intercorrelations Between Variables (Study 3)

Variable	*M*	*SD*	1	2	3
1. Endorsement of the antifemininity norm	3.45	1.70	–		
2. Legitimation of sexual harassment	5.52	1.63	.33**	–	
3. Willingness to intervene	3.76	1.01	-.48**	-.20*	–

*Note*. *p < .05, **p < .01

### Manipulation Check

The analysis revealed a significant main effect of the gender norm manipulation, *B* = 0.82, *t*(149) = 6.13, *p* < .001, 95% CI [0.55, 1.08], *η*^2^_p_ < .20. As expected, participants in the feminization condition (*M* = 4.58, *SE* = 0.19) perceived men as more feminized than those in the traditional masculinity condition (*M* = 2.95, *SE* = 0.19). No other effects were significant (*ps* > .395).

### Willingness to Intervene

The analysis revealed a significant main effect of antifemininity endorsement, *B* = −0.58, *t*(149) = −6.79, *p* < .001, 95% CI = [−0.75, −0.41], *η*^2^_p_ = .24, indicating that participants with stronger endorsement were less willing to intervene. However, neither the main effect of the gender norm manipulation, *B* = −0.05, *t*(149) = −0.57, *p* = .569, 95% CI = [-0.22, 0.12], *η*^2^_p_ < .01, nor the interaction between antifemininity and gender norm,* B* = −0.10, *t*(149) = −1.17, *p* = .242, 95% CI = [−0.27, 0.07], *η*^2^_p_ = .01, were significant.

### Legitimation of Sexual Harassment

**Fig. 2:**
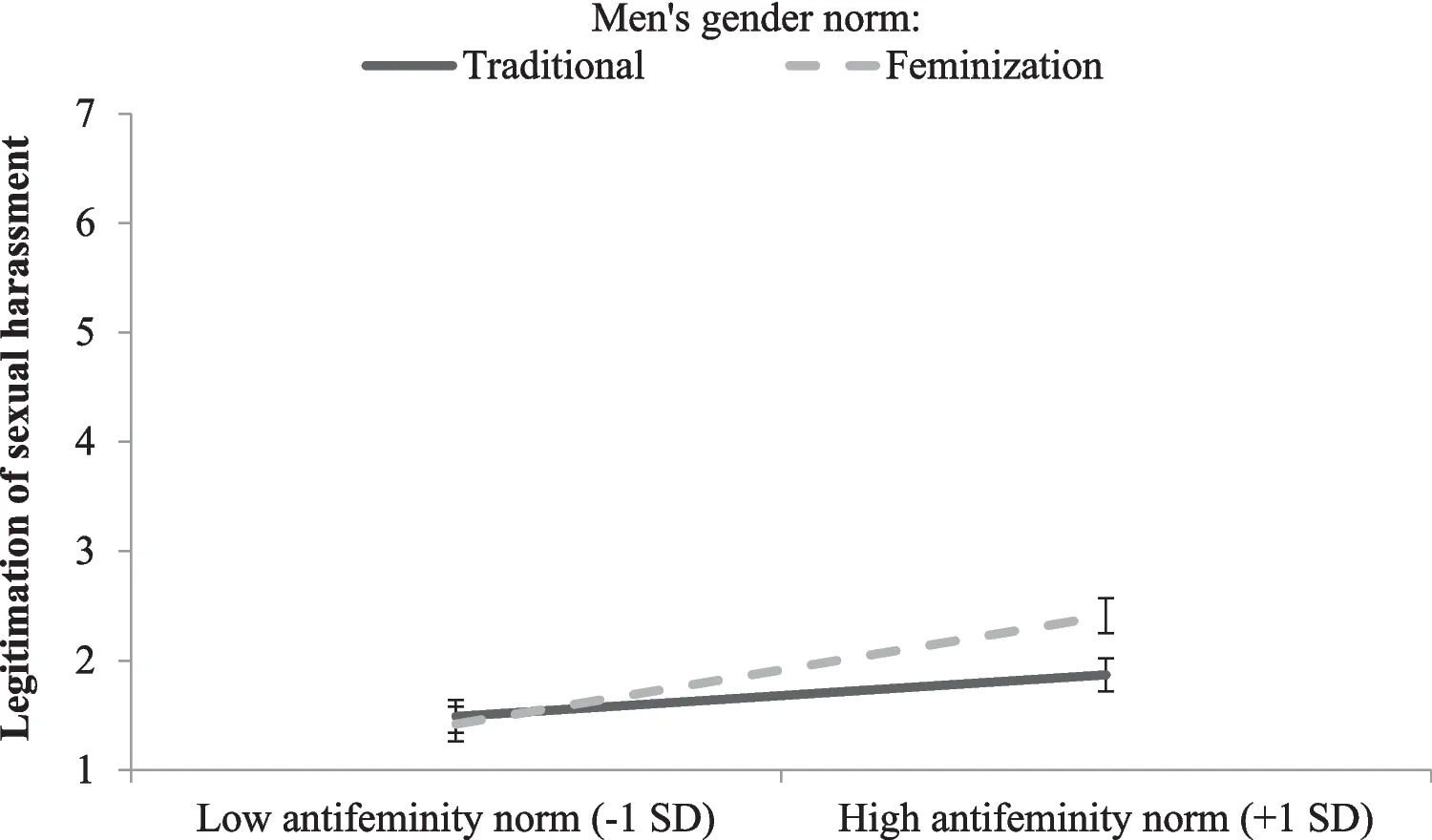
Legitimation of sexual harassment as a function of men’s gender norm and endorsement of the antifemininity norm (Study 3). Error bars are standard errors

The regression analysis revealed a significant main effect of antifemininity endorsement, *B* = 0.34, *t*(149) = 4.53, *p* < .001, 95% CI = [0.19, 0.49], *η*^2^_p_ = .12, such that stronger endorsement was associated with greater legitimation of sexual harassment. This effect was qualified by a significant interaction between gender norm and antifemininity endorsement, *B* = 0.15, *t*(149) = 2.03, *p* = .044, 95% CI = [0.00, 0.30], *η*^2^_p_ = .03 (see Fig. 2). Simple slope analysis showed that among men high in antifemininity (+ 1 *SD*), legitimation was greater in the feminization condition (*M* = 2.41, *SE* = 0.16) than in the traditional condition (*M* = 1.87, *SE* = 0.15),* B* = 0.27, *t*(149) = 2.53, *p* = .013, 95% CI = [0.59, 0.48], *η*^2^_p_ = .04. Among men low in antifemininity (−1 *SD*), the difference between conditions was not significant *B* = -0.04, *t*(149) = −0.36, *p* = .723, 95% CI = [−0.25, 0.17], *η*^2^_p_ < .01.

## Discussion

Results of Study 3 provided partial support for our hypotheses. Unlike Study 1, the interaction between men’s gender norm and endorsement of the antifemininity norm was not significant in predicting willingness to intervene. This null finding suggests that neither the conformity hypothesis (H1a) nor the resistance hypothesis (H1b) were supported for this outcome in Study 3. However, the predicted interaction emerged for legitimation of sexual harassment, offering support for H2. Consistent with the resistance hypothesis (H2b), men who strongly endorsed the antifemininity norm were more likely to legitimize sexual harassment in the feminization condition compared to the traditional condition. In contrast, men low in antifemininity endorsement did not differ across conditions, providing no support for the conformity hypothesis (H2a).

### Small-Scale Meta-Analysis

**Fig. 3:**
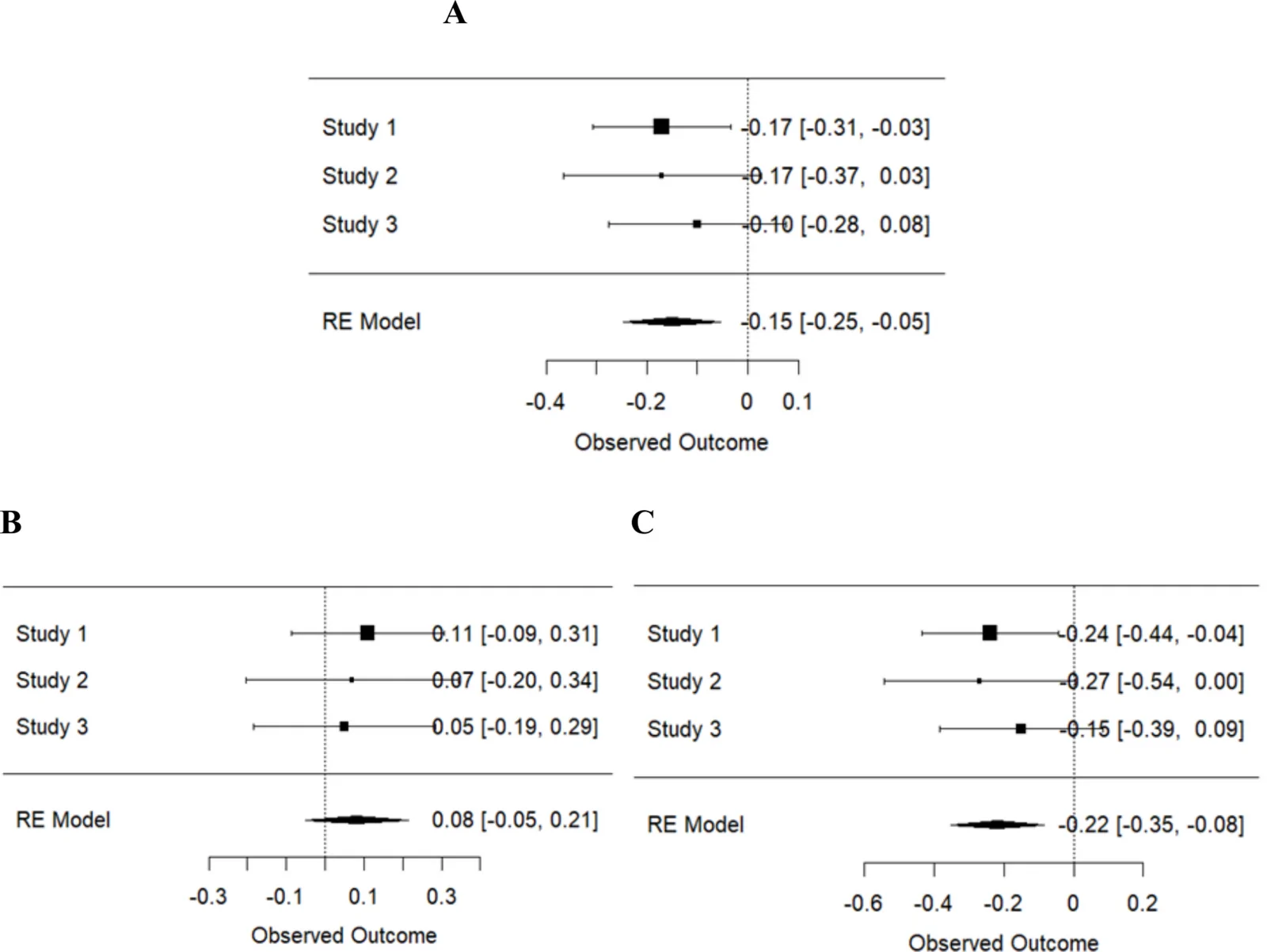
Forest plots from the random-effects meta-analyses: (**A**) Interaction between men’s gender norm and motivation to uphold the antifemininity norm on willingness to intervene, (**B**) simple effect of men’s gender norm among men low in motivation to uphold the antifemininity norm, and (**C**) simple effect of men’s gender norm among men high in motivation to uphold antifemininity norm

Across the three studies, the interaction between men’s gender norm manipulation and motivation to uphold antifemininity on willingness to intervene showed mixed results. To clarify these findings, we conducted a small-scale meta-analysis integrating data from all three studies (see Fig. 3). This approach allows for a more comprehensive evaluation of cumulative evidence, increased statistical power, and helps identifying consistent patterns across datasets (Goh et al., 2016).

We conducted random-effects meta-analyses using the *Metafor* package in R (Viechtbauer, 2010), applying the Sidik-Jonkman estimator, which is recommended when the number of studies is small (Inthout et al., 2014). The overall interaction between men’s gender norm and antifemininity was significant, *B* = -0.15, *SE* = 0.05, *z* = −3.05, *p* = .002, 95% CI [−0.25, −0.05], with non-significant heterogeneity, *T*^*2*^ = 0.00, *I*^*2*^ = 2.69%, and Q (*df* = 2) = 0.43, *p* = .806), indicating consistency across studies (see Fig. 3A). Analyses of simple effects did not support the conformity hypothesis (H1a): Among men low in motivation to uphold the antifemininity norm, the effect of men’s gender norm was not significant (*B* = 0.08, *p* = .225), with low heterogeneity (*T*^*2*^ = 0.01, *I*^*2*^ = 0.33%, and Q (*df* = 2) = 0.16, *p* = .925; see Fig. 3B). In contrast, findings consistently supported the resistance hypothesis (H1b). Among men high in antifemininity motivation, the effect of men’s gender norm was significant (*B* = -0.21, *SE* = 0.07, *z* = −3.18, *p* = .002, 95% CI [−0.35, −0.08]), with low heterogeneity (*T*^*2*^ = 0.02, *I*^*2*^ = 3.82%, and Q (*df* = 2) = 0.51, *p* = .776; see Fig. 3C). Taken together, the findings of the small-scale meta-analysis suggest that perceptions of men’s feminization—or broader shifts in masculinity norms—may reduce willingness to intervene, particularly among men strongly motivated to uphold the antifemininity norm.

## General Discussion

Across three studies, this research examined whether perceived changes in men’s gender norms influence heterosexual men’s willingness to intervene as active bystanders in workplace sexual harassment scenarios. Specifically, we investigated whether this effect is moderated by men’s motivation to uphold the antifemininity norm, a central component of traditional masculinity. Drawing on social role theory (Eagly, 1987), we hypothesized that perceiving men’s norms as becoming more feminine (vs. traditional) would increase willingness to intervene among men with low motivation to uphold the antifemininity norm (conformity dynamic, H1a). In contrast, based on precarious manhood theory (Vandello et al., 2008), we predicted that perceived feminization would trigger defensive responses, reducing intervention willingness among men with high motivation to protect the antifemininity norm (resistance dynamic, H1b). Results from the meta-analysis across the three studies provided empirical support for the resistance hypothesis.

Overall, these findings suggest that perceived shifts in gender norms may trigger defensive reactions among men invested in traditional masculinity, ultimately reducing their likelihood of acting as active bystanders. In contrast, men with lower motivation to uphold the antifemininity norm may already be more receptive to prosocial responses, regardless of perceived shifts in masculinity norms. Their weaker commitment to traditional masculinity likely contributes to a consistently higher willingness to intervene, leaving limited room for additional influence from the gender norm manipulation. Future research should explore whether conformity dynamics emerge when norm changes are framed as injunctive norms — that is, as explicit social expectations — potentially increasing their motivational relevance for men who are less invested in traditional gender roles.

In addition to assessing willingness to intervene, Studies 1 and 2 examined recognition of workplace sexual harassment. Both studies found that men strongly motivated to uphold the antifemininity norm were less likely to identify inappropriate behavior as harassment. This aligns with prior research linking traditional masculinity ideology to minimization and denial of harassment contexts (e.g., Bohner et al., 2006; Rebeiz & Harb, 2010). Study 3 extended these findings by examining harassment legitimation—specifically, the extent to which participants justified inappropriate behavior. Results aligned with past research (e.g., Chapleau et al., 2008; Suarez & Gadalla, 2010) revealing that men strongly motivated to uphold the antifemininity norm were more likely to legitimize harassment, reinforcing the role of deep-seated gender beliefs in shaping moral evaluations of workplace conduct.

Importantly, Study 3 also revealed a significant interaction effect: Men high in antifemininity endorsement were more likely to legitimize harassment in the feminization condition than in the traditional masculinity condition. This suggests that justification of harassment is not only rooted in personal beliefs but also influenced by situational cues—particularly how masculinity is defined within a given context. These findings underscore the importance of distinguishing between recognition and legitimation—two related but distinct processes shaped by both identity-relevant beliefs and contextual framing. To foster safer and more inclusive workplaces, interventions should go beyond raising awareness and actively challenge normative beliefs about masculinity.

### Limitations and Future Research Directions

This research provides valuable insights into how traditional masculinity influences men’s willingness to intervene in workplace sexual harassment, but several limitations warrant consideration. First, the low reliability of the willingness-to-intervene scale in Study 3 raises concerns about measurement validity. The scale was newly constructed for this study, with some items adapted to fit the experimental context. However, the lack of prior validation and potential issues in item wording—such as length or ambiguity—may have compromised internal consistency. Future research should focus on developing and validating a robust measure, ensuring clarity and coherence through pretesting and psychometric evaluation. However, this limitation may be tempered by the fact that, despite slight differences in measurement across studies, the results were highly consistent — as indicated in the meta-analysis by the non-significant degree of heterogeneity.

Second, the reliance on self-predicted behavior is a limitation. Participants were asked to imagine how they would respond in a hypothetical workplace sexual harassment scenario, which may not reflect real-world actions. Research shows that individuals often misjudge their future behavior due to cognitive biases and social desirability concerns. People tend to overestimate their likelihood of acting morally or courageously in imagined situations (Epley & Dunning, 2000; Van Bavel et al., 2015), and predicted behavior often diverges from actual behavior in emotionally charged or socially complex contexts (Baumeister et al., 2007). This gap is particularly relevant in bystander intervention research, where intentions do not always translate into action (Ajzen, 1991; Banyard et al., 2004). Future studies should incorporate behavioral or field-based measures to better capture intervention in real-world settings.

Third, features of the workplace sexual harassment scenario may have influenced responses in ways that limit generalizability. The harasser was portrayed as the direct superior of both the victim and the bystander, introducing a clear power differential. Prior research suggests harassment is more readily recognized when perpetrated by authority figures (Bursik, 1992; Cleveland & Kerst, 1993), but such power dynamics can also deter intervention (Casey & Ohler, 2012; McDonald et al., 2016). This hierarchical framing may have reduced participants’ willingness to intervene. Moreover, the use of a single vignette limits ecological validity. Future studies should test these dynamics across varied contexts and real-world situations to better understand how situational factors shape intervention.

Finally, sample homogeneity restricts the generalizability of our findings. Most participants were Caucasian, raising concerns about the applicability of results to more diverse populations. Research indicates that race and ethnicity shape perceptions of sexual violence, with men of color often facing disproportionate scrutiny and penalties (Harris & Grace, 1999; Jee-Lyn García & Sharif, 2015). Moreover, cultural and social experiences may influence how men from minority groups interpret and respond to harassment (Bowleg et al., 2016).

In addition to these concerns, we acknowledge the potential influence of demand characteristics. No formal assessment of participant suspicion was conducted during debriefing, which limits our ability to determine whether participants’ awareness of the study’s aims may have affected their responses. Future research should therefore prioritize both more inclusive sampling to capture intersectional dynamics and the inclusion of suspicion checks to better evaluate the impact of study awareness on participant behavior, thereby enhancing external validity.

### Clinical and Policy Implications

These findings have important implications for clinical practice and workplace policy. The tendency for men with strong antifemininity motivation to minimize or legitimize sexual harassment highlights the need for targeted interventions that address rigid gender norms. Clinically, masculinity ideology should be considered in therapeutic contexts, particularly when working with men who struggle with interpersonal boundaries or accountability in workplace dynamics. Psychoeducational programs that challenge traditional masculinity scripts and promote empathy may help reduce resistance to recognizing and intervening in harassment (Kaya et al., 2020).

From a policy perspective, organizations should recognize that adherence to traditional gender norms can undermine harassment prevention efforts. Training should move beyond legal definitions and procedural guidelines to address the cultural and psychological factors shaping perceptions of harassment. Policies should also account for power dynamics in workplace hierarchies (KelpHR, 2023), ensuring that employees feel empowered to intervene even when perpetrators hold authority.

## Conclusion

Guided by theories of gender role socialization and precarious manhood, this study examined how perceived shifts in masculinity norms influence men’s willingness to intervene in workplace sexual harassment. Findings revealed that perceiving masculinity as more feminine reduced intervention willingness among men strongly motivated to uphold antifemininity norms, underscoring the defensive dynamics triggered by challenges to traditional gender ideologies. These results extend prior research on bystander intervention by highlighting the role of identity-relevant beliefs in shaping responses to harassment and reinforce the importance of addressing cultural norms in prevention efforts. Beyond theoretical contributions, this work carries practical implications for clinical and organizational contexts: Interventions should incorporate strategies that challenge rigid masculinity norms and improve recognition of harassment, while organizational policies should extend beyond mere procedural compliance to actively confront internalized gender norms and beliefs that contribute to harassment. Future research should explore whether framing norm changes as injunctive expectations or integrating masculinity-focused training can enhance men’s engagement as active bystanders, ultimately fostering safer and more equitable work environments.

## Supplementary Information

Below is the link to the electronic supplementary material.

Supplementary file1 (DOCX 21 KB)

## Data Availability

All data supporting the findings of this study are openly available in the OSF repository at DOI: https://doi.org/10.17605/OSF.IO/F3R8U.

## References

[CR1] Ajzen, I. (1991). The theory of planned behavior. *Organizational Behavior and Human Decision Processes,**50*(2), 179–211. 10.1016/0749-5978(91)90020-T

[CR2] Andreasson, J., & Johansson, T. (2016). Global narratives of fatherhood: Fathering and masculinity on the internet. *International Review of Sociology,**26*(3), 482–496. 10.1080/03906701.2016.1191245

[CR3] Angelone, D. J., Mitchell, D., & Smith, D. (2018). The influence of gender ideology, victim resistance, and spiking a drink on acquaintance rape attributions. *Journal of Interpersonal Violence,**33*(20), 3186–3210. 10.1177/0886260516635318 26917569

[CR4] Babl, J. D. (1979). Compensatory masculine responding as a function of sex role. *Journal of Consulting and Clinical Psychology,**47*(2), 252–257. 10.1037/0022-006x.47.2.252 469071

[CR5] Baildon, A. E., Eagan, S. R., Checkalski, O. R., DiLillo, D., & Gervais, S. J. (2025). Associations between women’s and men’s conformity to gender norms and bystander intervention to disrupt sexual assault. *Sex Roles,**91*, Article 46. 10.1007/s11199-025-01597-z

[CR6] Banyard, V. L., & Moynihan, M. M. (2011). Variation in bystander behavior related to sexual and intimate partner violence prevention: Correlates in a sample of college students. *Psychology of Violence,**1*(4), 287–301. 10.1037/a0023544

[CR7] Banyard, V. L., Plante, E. G., & Moynihan, M. M. (2004). Bystander education: Bringing a broader community perspective to sexual violence prevention. *Journal of Community Psychology,**32*(1), 61–79. 10.1002/jcop.10078

[CR8] Banyard, V. L., Plante, G. E., & Moynihan, M. M. (2005). *Rape prevention through bystander education* [Final report to NIJ for grant 2002-WG-BX-0009]. https://www.ncjrs.gov/pdffiles1/nij/grants/208701.pdf

[CR9] Baumeister, R. F., Vohs, K. D., DeWall, C. N., & Zhang, L. (2007). How emotion shapes behavior: Feedback, anticipation, and reflection, rather than direct causation. *Personality and Social Psychology Review,**11*(2), 167–203. 10.1177/1088868307301033 18453461

[CR10] Bell, S. T., Kuriloff, P. J., & Lottes, I. (1994). Understanding attributions of blame in stranger rape and date rape situations: An examination of gender, race, identification, and students’ social perceptions of rape victims. *Journal of Applied Social Psychology,**24*(19), 1719–1734. 10.1111/j.1559-1816.1994.tb01571.x

[CR11] Bem, S. L. (1974). The measurement of psychological androgyny. *Journal of Consulting and Clinical Psychology,**42*(2), 155–162. 10.1037/h0036215 4823550

[CR12] Bennett, S., Banyard, V. L., & Garnhart, L. (2014). To act or not to act, that is the question? Barriers and facilitators of bystander intervention. *Journal of Interpersonal Violence,**29*(3), 476–496. 10.1177/0886260513505210 24097909

[CR13] Bohner, G., Siebler, F., & Schmelcher, J. (2006). Social norms and the likelihood of raping: Perceived rape myth acceptance of others affects men’s rape proclivity. *Personality and Social Psychology Bulletin,**32*(3), 286–297. 10.1177/0146167205280912 16455857

[CR14] Borinca, I., Iacoviello, V., & Valsecchi, G. (2021). Men’s discomfort and anticipated sexual misclassification due to counter-stereotypical behaviors: The interplay between traditional masculinity norms and perceived men’s femininization. *Sex Roles,**85*, 128–141. 10.1007/s11199-020-01210-5 PMC8550511 34776595

[CR15] Bosak, J., Eagly, A. H., Diekman, A., & Sczesny, S. (2018). Women and men of the past, present, and future: Evidence of dynamic gender stereotypes in Ghana. *Journal of Cross-Cultural Psychology,**49*(1), 115–129. 10.1177/0022022117738750

[CR16] Bosson, J. K., & Michniewicz, K. S. (2013). Gender dichotomization at the level of ingroup identity: What it is, and why men use it more than women. *Journal of Personality and Social Psychology,**105*(3), 425–442. 10.1037/a0033126 23750813

[CR17] Bosson, J. K., Vandello, J. A., Burnaford, R. M., Weaver, J. R., & Arzu Wasti, S. (2009). Precarious manhood and displays of physical aggression. *Personality and Social Psychology Bulletin,**35*(5), 623–634. 10.1177/0146167208331161 19202132

[CR18] Bowleg, L., English, D., del Rio-Gonzalez, A. M., Burkholder, G. J., Teti, M., & Tschann, J. M. (2016). Measuring the pros and cons of what it means to be a Black man: Development and validation of the Black Men’s Experiences Scale (BMES). *Psychology of Men & Masculinity,**17*(2), 177–188. 10.1037/men0000026 PMC4827438 27087786

[CR19] Brewster, M., & Tucker, J. M. (2016). Understanding bystander behavior: The influence of and interaction between bystander characteristics and situational factors. *Victims & Offenders,**11*(3), 455–481. 10.1080/15564886.2015.1009593

[CR20] Burn, S. M. (2009). A situational model of sexual assault prevention through bystander intervention. *Sex Roles,**60*, 779–792. 10.1007/s11199-008-9581-5

[CR21] Burns, M. D., Hamilton, A. N., & Paz Martin, S. (2024). “When men were men”: The effects of gender nostalgia on bias against trans men and women. *Psychology & Sexuality,**16*(1), 114–129. 10.1080/19419899.2024.2358229

[CR22] Bursik, K. (1992). Perceptions of sexual harassment in an academic context. *Sex Roles,**27*(7–8), 401–412. 10.1007/BF00289948

[CR23] Carlson, M. (2008). I’d rather go along and be considered a man: Masculinity and bystander intervention. *The Journal of Men’s Studies,**16*(1), 3–17. 10.3149/jms.1601.3

[CR24] Casey, E. A., & Ohler, K. (2012). Being a positive bystander: Male antiviolence allies’ experiences of “stepping up.” *Journal of Interpersonal Violence,**27*(1), 62–83. 10.1177/0886260511416479 PMC4270348 21859757

[CR25] Chapleau, K. M., Oswald, D. L., & Russell, B. L. (2008). Male rape myths: The role of gender, violence, and sexism. *Journal of Interpersonal Violence,**23*(5), 600–615. 10.1177/0886260507313529 18259049

[CR26] Chapleau, K. M. (2010). *The effect of victim status and system threat on rape myth acceptance* [Doctoral dissertation, Marquette University]. https://epublications.marquette.edu/dissertations_mu/61/

[CR27] Cheng, L., Hao, M., Xiao, L., & Wang, F. (2020). Join us: Dynamic norms encourage women to pursue STEM. *Current Psychology,**41*, 5967–5977. 10.1007/s12144-020-01105-4

[CR28] Christofidou, A. (2021). Men and masculinities: A continuing debate on change. *Norma,**16*(2), 81–97. 10.1080/18902138.2021.1891758

[CR29] Cleveland, J. N., & Kerst, M. E. (1993). Sexual harassment and perceptions of power: An under-articulated relationship. *Journal of Vocational Behavior,**42*(1), 49–67. 10.1006/jvbe.1993.1004

[CR30] Cole, B. P., Brennan, M., Tyler, E., & Willard, R. (2020). Predicting men’s acceptance of sexual violence myths through conformity to masculine norms, sexism, and “locker room talk.” *Psychology of Men & Masculinities,**21*(4), 508–517. 10.1037/men0000248

[CR31] Connell, R. W., & Messerschmidt, J. W. (2005). Hegemonic masculinity: Rethinking the concept. *Gender & Society,**19*(6), 829–859. 10.1177/0891243205278639

[CR32] Connell, R. W. (2005). *Masculinities* (2nd ed.). 10.4324/9781003116479

[CR33] Croft, A., Atkinson, C., Sandstrom, G., Orbell, S., & Aknin, L. (2020). Loosening the GRIP (Gender Roles Inhibiting Prosociality) to promote gender equality. *Personality and Social Psychology Review,**25*(1), 66–92. 10.1177/1088868320964615 33427043

[CR34] de Boise, S. (2022). Changing men, changing masculinities. *NORMA: International Journal for Masculinity Studies,**17*(4), 213–218. 10.1080/18902138.2022.2133819

[CR35] Diekman, A. B., & Eagly, A. H. (2000). Stereotypes as dynamic constructs: Women and men of the past, present, and future. *Personality and Social Psychology Bulletin,**26*(10), 1171–1188. 10.1177/0146167200262001

[CR36] Diekman, A. B., & Goodfriend, W. (2006). Rolling with the changes: A role congruity perspective on gender norms. *Psychology of Women Quarterly,**30*(4), 369–383. 10.1111/j.1471-6402.2006.00312.x

[CR37] Eagly, A. H., & Wood, W. (2012). Social role theory. In P. A. M. Van Lange, A. W. Kruglanski, & E. T. Higgins (Eds.), *Handbook of theories of social psychology* (Vol. 2, pp. 458–476). Sage Publications Ltd. 10.4135/9781446249222.n49

[CR38] Eagly, A. H. (1987). *Sex differences in social behavior: A social-role interpretation* (1st ed.). Psychology Press. 10.4324/9780203781906

[CR39] Eisen, D. B., & Yamashita, L. (2019). Borrowing from femininity: The caring man, hybrid masculinities, and maintaining male dominance. *Men and Masculinities,**22*(5), 801–820. 10.1177/1097184X17728552

[CR40] Elliott, K. (2016). Caring masculinities: Theorizing an emerging concept. *Men and Masculinities,**19*(3), 240–259. 10.1177/1097184X15576203

[CR41] Epley, N., & Dunning, D. (2000). Feeling “holier than thou”: Are self-serving assessments produced by errors in self- or social prediction? *Journal of Personality and Social Psychology,**79*(6), 861–875. 10.1037/0022-3514.79.6.861 11138757

[CR42] Fabiano, P. M., Perkins, W., Berkowitz, A., Linkenbach, J., & Stark, C. (2003). Engaging men as social justice allies in ending violence against women: Evidence for a social norms approach. *Journal of American College Health,**52*(3), 105–112. 10.1080/07448480309595732 14992295

[CR43] Falomir-Pichastor, J. M., Berent, J., & Anderson, J. (2019). Perceived men’s feminization and attitudes toward homosexuality: Heterosexual men’s reactions to the decline of the anti-femininity norm of masculinity. *Sex Roles,**81*, 208–222. 10.1007/s11199-018-0985-6

[CR44] Flood, M. (2020). *Who is a real man? Most Australians believe outdated ideals of masculinity are holding men back*. The Conversation*.* https://theconversation.com/who-is-a-real-man-most-australians-believe-outdated-ideals-of-masculinity-are-holding-men-back-147847

[CR45] Franklin, C. A., Brady, P. Q., & Ashworth, L. M. (2020). Bystander behaviors following an accusation of rape: The effect of relational distance and participant sex on victim-centered intervention. *Journal of School Violence,**19*(3), 292–308. 10.1080/15388220.2019.1680558

[CR46] Froehlich, L., Olsson, M. I. T., Dorrough, A. R., & Martiny, S. E. (2020). Gender at work across nations: Men and women working in male-dominated and female-dominated occupations are differentially associated with agency and communion. *Journal of Social Issues,**76*, 484–511. 10.1111/josi.12390

[CR47] Glick, P., Gangl, C., Gibb, S., Klumpner, S., & Weinberg, E. (2007). Defensive reactions to masculinity threat: More negative affect toward effeminate (but not masculine) gay men. *Sex Roles,**57*(1–2), 55–59. 10.1007/s11199-007-9195-3

[CR48] Goh, J. X., Hall, J. A., & Rosenthal, R. (2016). Mini meta-analysis of your own studies: Some arguments on why and a primer on how. *Social and Personality Psychology Compass,**10*(10), 535–549. 10.1111/spc3.12267

[CR49] Good, J. J., Moss-Racusin, C. A., & Sanchez, D. T. (2018). When do we intervene? The role of gender norms and bystander behavior. *Journal of Social Issues,**74*(4), 894–911. 10.1111/josi.12299

[CR50] GQ. (2019). *The state of masculinity now: A GQ survey*. https://www.gq.com/story/state-of-masculinity-survey

[CR51] Gray, J. M. (2006). Rape myth beliefs and prejudiced instructions: Effects on decisions of guilt in a case of date rape. *Legal and Criminological Psychology,**11*(1), 75–80. 10.1348/135532505X68250

[CR52] Handy, J. (2006). Sexual harassment in small-town New Zealand: A qualitative study of three contrasting organizations. *Gender, Work and Organization,**13*(1), 1–24. 10.1111/j.1468-0432.2006.00293.x

[CR53] Harris, A. J., & Grace, K. T. (1999). Race and rape: A comparison of White and African American attitudes toward rape. *Journal of Black Studies,**29*(4), 559–575. 10.1177/002193479902900406

[CR54] Iacoviello, V., Valsecchi, G., Berent, J., Anderson, J., & Falomir-Pichastor, J. M. (2020). Heterosexual men’s attitudes towards homosexuality and ingroup distinctiveness: The role of perceived men’s feminisation. *Psychology & Sexuality,**11*(1–2), 45–61. 10.1080/19419899.2019.1675749

[CR55] Iacoviello, V., Valsecchi, G., Berent, J., Borinca, I., & Falomir -Pichastor, J. M. (2021). The impact of masculinity beliefs and political ideologies on men’s backlash against non-traditional men: The moderating role of perceived men’s feminization. *International Review of Social Psychology,**34*(1), Article 28. 10.5334/irsp.588

[CR56] Iacoviello, V., Valsecchi, G., Vétois, M., & Falomir-Pichastor, J. M. (2024). Reducing the gender gap on adolescents’ interest in study fields: The impact of perceived changes in ingroup gender norms and gender prototypicality. *Social Psychology of Education,**27*, 1043–1063. 10.1007/s11218-024-09909-z PMC11271416 39070575

[CR57] Ilies, R., Hauserman, N., Schwochau, S., & Stibal, J. (2003). Reported incidence rates of work-related sexual harassment in the United States: Using meta-analysis to explain reported rate disparities. *Personnel Psychology,**56*(3), 607–631. 10.1111/j.1744-6570.2003.tb00752.x

[CR58] Inthout, J., Ioannidis, J. P., & Borm, G. F. (2014). The Hartung-Knapp-Sidik-Jonkman method for random effects meta-analysis. *BMC Medical Research Methodology,**14*(1), Article 25. 10.1186/1471-2288-14-25 PMC4015721 24548571

[CR59] Jacobson, R. K., & Eaton, A. A. (2018). How organizational policies influence bystander likelihood of reporting moderate and severe sexual harassment at work. *Employee Responsibilities and Rights Journal,**30*(1), 37–62. 10.1007/s10672-017-9309-1

[CR60] Jee-Lyn García, J., & Sharif, M. Z. (2015). Black lives matter: A commentary on racism and public health. *American Journal of Public Health,**105*(8), e27–e30. 10.2105/AJPH.2015.302706 PMC4504294 26066958

[CR61] Johansson, T., & Andreasson, J. (2017). *Fatherhood in transition: Masculinity, identity and every-day life*. Palgrave Macmillan.

[CR62] Katz, J. (2006). *The macho paradox: Why some men hurt women and how all men can help*. Sourcebooks.

[CR63] Kaya, A., Le, T. P., Brady, J., & Iwamoto, D. (2020). Men who intervene to prevent sexual assault: A grounded theory study on the role of masculinity in bystander intervention. *Psychology of Men & Masculinities,**21*(3), 463–478. 10.1037/men0000249

[CR64] KelpHR. (2023). *Unveiling the power dynamics: Exploring the connection between power and sexual harassment in the workplace*. Kelp Blog. https://www.kelphr.com/blogs/unveiling-the-power-dynamics-exploring-the-connection-between-power-and-sexual-harassment-in-the-workplace/

[CR65] Kosakowska-Berezecka, N., Besta, T., Adamska, K., Jaśkiewicz, M., Jurek, P., & Vandello, J. A. (2016). If my masculinity is threatened I won’t support gender equality? The role of agentic self-stereotyping in restoration of manhood and perception of gender relations. *Psychology of Men & Masculinity,**17*(3), 274–284. 10.1037/men0000016

[CR66] Kroeper, K. M., Sanchez, D. T., & Himmelstein, M. S. (2014). Heterosexual men’s confrontation of sexual prejudice: The role of precarious manhood. *Sex Roles,**70*(1–2), 1–13. 10.1007/s11199-013-0306-z

[CR67] Leone, R. M., & Parrott, D. J. (2019). Misogynistic peers, masculinity, and bystander intervention for sexual aggression: Is it really just “locker-room talk?” *Aggressive Behavior,**45*, 42–51. 10.1002/ab.21795 30239007

[CR68] Leone, R. M., Parrott, D. J., Swartout, K. M., & Tharp, A. T. (2016). Masculinity and bystander attitudes: Moderating effects of masculine gender role stress. *Psychology of Violence,**6*(1), 82–90. 10.1037/a0038926 PMC5868745 29593932

[CR69] Leone, R. M., Parrott, D. J., & Swartout, K. M. (2017). When is it “manly” to intervene? Examining the effects of a misogynistic peer norm on bystander intervention for sexual aggression. *Psychology of Violence,**7*(2), 286–295. 10.1037/vio0000055

[CR70] Levant, R. F., Rankin, T. J., Williams, C. M., Hasan, N. T., & Smalley, K. B. (2010). Evaluation of the factor structure and construct validity of scores on the Male Role Norms Inventory—Revised (MRNI-R). *Psychology of Men & Masculinity,**11*(1), 25–37. 10.1037/a0017637

[CR71] Levant, R. F., Hall, R. J., & Rankin, T. J. (2013). Male Role Norms Inventory-Short Form (MRNI-SF): Development, confirmatory factor analytic investigation of structure, and measurement invariance across gender. *Journal of Counseling Psychology,**60*(2), 228–238. 10.1037/a0031545 23421776

[CR72] Levant, R. F., Hall, R. J., Weigold, I. K., & McCurdy, E. R. (2016). Construct validity evidence for the Male Role Norms Inventory-Short Form: A structural equation modeling approach using the bifactor model. *Journal of Counseling Psychology,**63*(5), 534–542. 10.1037/cou0000171 27598043

[CR73] Lonsway, K. A., & Fitzgerald, L. F. (1995). Attitudinal antecedents of rape myth acceptance: A theoretical and empirical reexamination. *Journal of Personality and Social Psychology,**68*(4), 704–711. 10.1037/0022-3514.68.4.704

[CR74] Maass, A., Cadinu, M., Guarnieri, G., & Grasselli, A. (2003). Sexual harassment under social identity threat: The computer harassment paradigm. *Journal of Personality and Social Psychology,**85*(5), 853–870. 10.1037/0022-3514.85.5.853 14599249

[CR75] Mainwaring, C., Gabbert, F., & Scott, A. J. (2022). A systematic review exploring variables related to bystander intervention in sexual violence contexts. *Trauma, Violence, & Abuse,**24*(3), 1727–1742. 10.1177/15248380221079660 PMC10240636 35343337

[CR76] McDermott, R. C., Levant, R. F., Hammer, J. H., Hall, R. J., McKelvey, D. K., & Jones, Z. (2017). Further examination of the factor structure of the Male Role Norms Inventory-Short Form (MRNI-SF): Measurement considerations for women, men of color, and gay men. *Journal of Counseling Psychology,**64*(6), 724–738. 10.1037/cou0000225 28541058

[CR77] McDonald, P., Charlesworth, S., & Graham, T. (2016). Action or inaction: Bystander intervention in workplace sexual harassment. *The International Journal of Human Resource Management,**27*(5), 548–566. 10.1080/09585192.2015.1023331

[CR78] Mills, J. S., Manea, A., & McCreary, D. R. (2023). Precarious manhood manifests as drive for muscularity among men. *Sex Roles,**88*(11–12), 547–555. 10.1007/s11199-023-01369-7

[CR79] Parent, M. C., & Moradi, B. (2009). Confirmatory factor analysis of the Conformity to Masculine Norms Inventory and development of the Conformity to Masculine Norms Inventory-46. *Psychology of Men & Masculinity,**10*(3), 175–189. 10.1037/a0015481

[CR80] Paterson, W., & Mahalik, J. R. (2024). *Masculine norm conformity, antifemininity, and heteronormativity: Unpacking their role in men’s aggression and the violation of women’s human rights*. Boston College Center for Human Rights and International Justice.

[CR81] Payne, D. L., Lonsway, K. A., & Fitzgerald, L. F. (1999). Rape myth acceptance: Exploration of its structure and its measurement using the Illinois Rape Myth Acceptance Scale. *Journal of Research in Personality,**33*(1), 27–68. 10.1006/jrpe.1998.2238

[CR82] Powell, A. (2011). *Review of bystander approaches in support of preventing violence against women*. Victorian Health Promotion Foundation. https://anrows.intersearch.com.au/anrowsjspui/handle/1/18912

[CR83] Preacher, K. J., Curran, P. J., & Bauer, D. J. (2006). Computational tools for probing interactions in multiple linear regression, multilevel modeling, and latent curve analysis. *Journal of Educational and Behavioral Statistics,**31*(4), 437–448. 10.3102/10769986031004437

[CR84] Rebeiz, M., & Harb, C. (2010). Perceptions of sexual harassment in the Lebanese workplace: Prevalence and psychological outcomes. *Journal of Interpersonal Violence,**25*(5), 874–886. 10.1177/0886260509336963

[CR85] Rivera-Rodriguez, A., Larsen, G., & Dasgupta, N. (2022). Changing public opinion about gender activates group threat and opposition to feminist social movements among men. *Group Processes & Intergroup Relations,**25*(3), 811–829. 10.1177/13684302211048885

[CR86] Rizzo, A. J., Banyard, V. L., & Edwards, K. M. (2021). Unpacking adolescent masculinity: Relations between boys’ sexual harassment victimization, perpetration, and gender role beliefs. *Journal of Family Violence,**36*, 825–835. 10.1007/s10896-020-00187-9

[CR87] Roberts, S. (2017). Domestic labour, masculinity and social change: Insights from working-class young men’s transitions to adulthood. *Journal of Gender Studies,**27*(3), 274–287. 10.1080/09589236.2017.1391688

[CR88] Salin, D. (2011). The significance of gender for third parties’ perceptions of negative interpersonal behaviour: Labelling and explaining negative acts. *Gender, Work & Organization,**18*(6), 571–591. 10.1111/j.1468-0432.2009.00465.x

[CR89] Savage, M. W., Menegatos, L., & Roberto, A. J. (2017). When do friends prevent friends from hooking up intoxicated? An examination of sex differences and hypothetical intoxication in peer interventions. *Archives of Sexual Behavior,**46*(6), 1819–1829. 10.1007/s10508-017-0969-6 28474308

[CR90] Schmitt, M. T., & Branscombe, N. R. (2001). The good, the bad, and the manly: Threats to one’s prototypicality and evaluations of fellow in-group members. *Journal of Experimental Social Psychology,**37*(5), 364–376. 10.1006/jesp.2001.1476

[CR91] Schneider, M., Moriarty, T., & Gough, B. (2009). Understanding attribution of blame in cases of rape: An analysis of participant gender, type of rape and perceived similarity to the victim. *Journal of Sexual Aggression,**15*(1), 63–81. 10.1080/13552600802641649

[CR92] Simmons, J. P., Nelson, L. D., & Simonsohn, U. (2011). False-positive psychology: Undisclosed flexibility in data collection and analysis allows presenting anything as significant. *Psychological Science,**22*(11), 1359–1366. 10.1177/0956797611417632 22006061

[CR93] Simmons, J. P., Nelson, L. D., & Simonsohn, U. (2013). *Life after p-hacking* [Conference presentation]. Annual meeting of the Society for Personality and Social Psychology, New Orleans, LA. 10.2139/ssrn.2205186

[CR94] Stewart, A. (2014). Alternative masculinities and the challenge of relational responsibility. In À. Carabí & J. M. Armengol (Eds.), *Alternative masculinities for a changing world* (pp. 217–230). Palgrave Macmillan. 10.1057/9781137462565

[CR95] Suarez, E., & Gadalla, T. M. (2010). Stop blaming the victim: A meta-analysis on rape myths. *Journal of Interpersonal Violence,**25*(11), 2010–2035. 10.1177/0886260509354503 20065313

[CR96] Thompson, E. H., & Bennett, K. M. (2015). Measurement of masculinity ideologies: A (critical) review. *Psychology of Men & Masculinity,**16*, 115–133. 10.1037/a0038609

[CR97] Timmerman, G., & Bajema, C. (1999). Sexual harassment in Northwest Europe. *European Journal of Women’s Studies,**6*(4), 419–439. 10.1177/135050689900600402

[CR98] Valsecchi, G., Iacoviello, V., Berent, J., Anderson, J. R., Borinca, I., & Falomir-Pichastor, J. M. (2022). Gay-gender expression and attitudes toward gay people: The moderating role of perceived men’s feminization. *Psychology of Sexual Orientation and Gender Diversity,**9*(1), 120–126. 10.1037/sgd0000452

[CR99] Valsecchi, G., Iacoviello, V., Berent, J., Borinca, I., & Falomir-Pichastor, J. M. (2023). Men’s gender norms and gender-hierarchy-legitimizing ideologies: The effect of priming traditional masculinity versus a feminization of men’s norms. *Gender Issues,**40*(2), 145–167. 10.1007/s12147-022-09308-8 PMC10689301 38044965

[CR100] Van Bavel, J. J., Packer, D. J., Haas, I. J., & Cunningham, W. A. (2015). The neuroscience of moral cognition: From dual-process models to dynamic systems. *Current Opinion in Psychology,**6*, 167–172. 10.1016/j.copsyc.2015.08.008

[CR101] Vandello, J. A., & Cohen, D. (2008). Culture, gender, and men’s intimate partner violence. *Social and Personality Psychology Compass,**2*(2), 652–667. 10.1111/j.1751-9004.2008.00080.x

[CR102] Vandello, J. A., Bosson, J. K., Cohen, D., Burnaford, R. M., & Weaver, J. R. (2008). Precarious manhood. *Journal of Personality and Social Psychology,**95*(6), 1325–1339. 10.1037/a0012453 19025286

[CR103] Vandello, J. A., Hettinger, V. E., Bosson, J. K., & Siddiqi, J. (2013). When equal isn’t really equal: The masculine dilemma of seeking work flexibility. *Journal of Social Issues,**69*(2), 303–321. 10.1111/josi.12016

[CR104] Vescio, T. K., Schermerhorn, N. E. C., Lewis, K. A., Yamaguchi-Pedroza, K., & Loviscky, A. J. (2023). Masculinity threats sequentially arouse public discomfort, anger, and positive attitudes toward sexual violence. *Personality and Social Psychology Bulletin,**51*(1), 96–109. 10.1177/01461672231179431 PMC11616216 37417681

[CR105] Viechtbauer, W. (2010). Conducting meta-analyses in R with the metafor package. *Journal of Statistical Software, 36*(3). 10.18637/jss.v036.i03

[CR106] Wall, K., Aboim, S., & Marinho, S. (2007). Fatherhood, family and work in men’s lives: Negotiating new and old masculinities. *Recherches Sociologiques Et Anthropologiques,**38*(2), 105–122. 10.4000/rsa.470

[CR107] Warren, T. (2022). Work-time, male-breadwinning and the division of domestic labour: Male part-time and full-time workers in unsettled times. *Sociology,**56*(1), 72–96. 10.1177/00380385211019660

[CR108] Wester, S. R., Vogel, D. L., O’Neil, J. M., & Danforth, L. (2012). Development and evaluation of the Gender Role Conflict Scale Short Form (GRCS-SF). *Psychology of Men & Masculinity,**13*(2), 199–210. 10.1037/a0025550 PMC4500044 26185486

[CR109] Willness, C. R., Steel, P., & Lee, K. (2007). A meta-analysis of the antecedents and consequences of workplace sexual harassment. *Personnel Psychology,**60*(1), 127–162. 10.1111/j.1744-6570.2007.00067.x

[CR110] Wolfman, G., Hearn, J., & Yeadon-Lee, T. (2021). Hollow femininities: The emerging faces of neoliberal masculinities. *NORMA: International Journal for Masculinity Studies,**16*(4), 217–234. 10.1080/18902138.2021.1996829

[CR111] Wong, Y. J., & Wang, S.-Y. (2022). Toward an integrative psychology of masculinities. *Psychology of Men & Masculinities,**23*(3), 285–298. 10.1037/men0000387

