# Supplementary material for: Evolving Masculinity Norms Shape Men’s Willingness to Intervene in Workplace Sexual Harassment
Source: Sex Roles. 2026 Feb 5;92(1):3. doi: 10.1007/s11199-025-01635-w (PMC13652909; doi:10.1007/s11199-025-01635-w)
Supplement: Supplementary file 1 [file 11199_2025_1635_MOESM1_ESM.docx]

**Online Supplements**

**Table S1**

*Preliminary Tests for the Exploratory Factor Analysis of the Willingness to Intervene Scale (Study 1)*

| **Test** | Result |
| --- | --- |
| Bartlett's Test of Sphericity | χ² (10) = 244.29, *p* < .001 |
| Kaiser-Meyer-Olkin (KMO) measure | Overall MSA = 0.77 |
| KMO per item | W1 = 0.83, W2 = 0.75, W3 = 0.80, W4 = 0.75, W5 = 0.75 |

**Table S2**

*Descriptive Statistics and Exploratory Factor Analysis Loadings for the Willingness to Intervene Scale (Study 1)*

| Item | *M* | *SD* | Loading | Communalities (h²) | Uniqueness (u²) |
| --- | --- | --- | --- | --- | --- |
| I would talk to my boss about the incident. | 4.03 | 1.80 | 0.54 | 0.29 | 0.71 |
| I would contact human resources and report the incident. | 4.34 | 1.82 | 0.72 | 0.52 | 0.48 |
| I would go see my female colleague and ask her whether she wants to talk about the incident. | 5.55 | 1.43 | 0.55 | 0.30 | 0.70 |
| I would refrain from taking any specific action and act as though I hadn't witnessed anything. | 3.41 | 1.56 | 0.65 | 0.42 | 0.58 |
| I would organize a round table discussion at our workplace about sexual harassment and sexism. | 2.93 | 1.79 | 0.57 | 0.32 | 0.68 |

**Table S3**

*Results of Parallel Analysis and the Minimum Average Partial (MAP) test for Determining the Optimal Number of Factors for the Willingness to Intervene Scale (Study 1)*

| Factor | Observed Eigenvalue | Simulated Eigenvalue | Retained/Not retained | MAP Value | Final Decision |
| --- | --- | --- | --- | --- | --- |
| 1 | 2.47 | 1.10 | Yes | 0.072 | Retain |
| 2 | 0.81 | 1.03 | No | 0.181 | Do not retain |
| 3 | 0.69 | 0.95 | No | 0.404 | Do not retain |
| 4 | 0.57 | 0.88 | No | 1.0 | Do not retain |
| 5 | 0.46 | 0.75 | No | NA | Do not retain |

***Note.*** Parallel Analysis compares observed and simulated eigenvalues, retaining factors whose observed values exceed the simulated ones. The Minimum Average Partial (MAP) test identifies the number of factors associated with the lowest average partial correlation. Both methods supported a one-factor solution.
NA = Not Applicable.

**Table S4**

*Preliminary Tests for the Exploratory Factor Analysis of the Willingness to Intervene Scale (Study 2)*

| **Test** | Result |
| --- | --- |
| Bartlett's Test of Sphericity | χ² (10) = 359.84, *p* < .001 |
| Kaiser-Meyer-Olkin (KMO) measure | Overall MSA = 0.79 |
| KMO per item | W1 = 0.83, W2 = 0.78, W3 = 0.84, W4 = 0.76, W5 = 0.76 |

**Table S5**

*Descriptive Statistics and Exploratory Factor Analysis Loadings for the Willingness to Intervene Scale (Study 2)*

| Item | *M* | *SD* | Loading | Communalities (h²) | Uniqueness (u²) |
| --- | --- | --- | --- | --- | --- |
| I would talk to my boss about the incident. | 3.71 | 1.99 | 0.66 | 0.43 | 0.57 |
| I would contact human resources and report the incident. | 4.13 | 2.10 | 0.80 | 0.63 | 0.37 |
| I would go see my female colleague and ask her whether she wants to talk about the incident. | 4.87 | 1.92 | 0.62 | 0.39 | 0.61 |
| I would refrain from taking any specific action and act as though I hadn't witnessed anything. | 3.17 | 1.88 | 0.73 | 0.53 | 0.47 |
| I would organize a round table discussion at our workplace about sexual harassment and sexism. | 2.93 | 1.84 | 0.63 | 0.40 | 0.60 |

**Table S6**

*Results of Parallel Analysis and the Minimum Average Partial (MAP) test for Determining the Optimal Number of Factors for the Willingness to Intervene Scale (Study 2)*

| Factor | Observed Eigenvalue | Simulated Eigenvalue | Retained/Not retained | MAP Value | Final Decision |
| --- | --- | --- | --- | --- | --- |
| 1 | 2.90 | 1.12 | Yes | 0.083 | Retain |
| 2 | 0.76 | 1.03 | No | 0.183 | Do not retain |
| 3 | 0.52 | 0.95 | No | 0.402 | Do not retain |
| 4 | 0.50 | 0.84 | No | 1.0 | Do not retain |
| 5 | 0.32 | 0.74 | No | NA | Do not retain |

***Note.*** Parallel Analysis compares observed and simulated eigenvalues, retaining factors whose observed values exceed the simulated ones. The Minimum Average Partial (MAP) test identifies the number of factors associated with the lowest average partial correlation. Both methods supported a one-factor solution.
NA = Not Applicable.

**Table S7**

*Preliminary Tests for the Exploratory Factor Analysis of the Willingness to Intervene Scale (Study 3)*

| **Test** | Result |
| --- | --- |
| Bartlett's Test of Sphericity | χ² (10) = 88.69, *p* < .001 |
| Kaiser-Meyer-Olkin (KMO) measure | Overall MSA = 0.70 |

**Table S8**

*Descriptive Statistics and Exploratory Factor Analysis Loadings for the Willingness to Intervene Scale (Study 3)*

| Item | *M* | *SD* | Loading | Communalities (h²) | Uniqueness (u²) |
| --- | --- | --- | --- | --- | --- |
| I would talk to my boss about the incident. | 3.67 | 1.88 | 0.64 | 0.41 | 0.59 |
| I would contact human resources and report the incident. | 4.30 | 1.83 | 0.59 | 0.34 | 0.66 |
| I would go see my female colleague and ask her whether she wants to talk about the incident. | 5.84 | 1.22 | 0.41 | 0.17 | 0.83 |
| I would organize a round table discussion at our workplace about sexual harassment and sexism. | 4.14 | 1.74 | 0.68 | 0.46 | 0.54 |

**Table S9**

*Results of Parallel Analysis and the Minimum Average Partial (MAP) test for Determining the Optimal Number of Factors for the Willingness to Intervene Scale (Study 3)*

| Factor | Observed Eigenvalue | Simulated Eigenvalue | Retained/Not retained | MAP Value | Final Decision |
| --- | --- | --- | --- | --- | --- |
| 1 | 2.01 | 1.11 | Yes | 0.116 | Retain |
| 2 | 0.85 | 0.99 | No | 0.377 | Do not retain |
| 3 | 0.59 | 0.87 | No | 1.0 | Do not retain |
| 4 | 0.55 | 0.74 | No | NA | Do not retain |

Parallel Analysis compares observed and simulated eigenvalues, retaining factors whose observed values exceed the simulated ones. The Minimum Average Partial (MAP) test identifies the number of factors associated with the lowest average partial correlation. Both methods supported a one-factor solution.
NA = Not Applicable.
